# New Methodologies for Conducting Maternal, Infant, and Child Nutrition Research in the Era of COVID-19

**DOI:** 10.3390/nu13030941

**Published:** 2021-03-15

**Authors:** Jacqueline F. Gould, Karen Best, Merryn J. Netting, Robert A. Gibson, Maria Makrides

**Affiliations:** 1 Women and Kids, South Australian Health and Medical Research Institute, 72 King William Road, Adelaide 5006, Australia; jacqueline.gould@sahmri.com (J.F.G.); karen.best@sahmri.com (K.B.); merryn.netting@sahmri.com (M.J.N.); Robert.gibson@adelaide.edu.au (R.A.G.); 2 Discipline of Paediatrics, Faculty of Health and Medical Sciences, The University of Adelaide, North Terrace, Adelaide 5000, Australia; 3 Faculty of Health and Medical Sciences, School of Psychology, The University of Adelaide, North Terrace, Adelaide 5000, Australia; 4 Women’s and Children’s Hospital, 72 King William Road, Adelaide 5006, Australia

**Keywords:** clinical trial, COVID-19, coronavirus, pandemic, methodology, RCT, research methods

## Abstract

The severe acute respiratory syndrome coronavirus disease 2019 (COVID-19) outbreak rapidly became a worldwide pandemic in early 2020. In Australia, government-mandated restrictions on non-essential face-to-face contact in the healthcare setting have been crucial for limiting opportunities for COVID-19 transmission, but they have severely limited, and even halted, many research activities. Our institute’s research practices in the vulnerable populations of pregnant women and young infants needed to adapt in order to continue without exposing participants, or staff, to an increased risk of exposure to COVID-19. Here, we discuss our pre-and-post COVID-19 methods for conducting research regarding nutrition during pregnancy, infancy, and early childhood. We discuss modifications to study methods implemented to avoid face-to-face contact when identifying and recruiting potential participants, gaining informed consent, conducting appointments, and collecting outcome data, and the implications of these changes. The COVID-19 pandemic has required numerous changes to the conduct of research activities, but many of those modifications will be useful in post-COVID-19 research settings.

## 1. Introduction

The first 1000 days (conception through to 24 months of age) of a child’s life represent a key period of development, with lasting implications for lifelong growth, health, and neurodevelopment. Nutrition during this time is considered to be the most influential (non-genetic) determinant of development [1,2,3,4,5,6] and hence is the focus of much research that seeks to optimize health and development [7,8,9,10].

The recruitment of participants for our observational dietary studies and nutritional intervention trials within the first 1000 days has in the past been conducted through direct person-to-person approach to families in clinical departments of health care services. Participation in studies has typically involved face-to-face appointments for consent, enrollment, and data collection procedures.

The severe acute respiratory syndrome coronavirus disease 2019 (COVID-19) outbreak was initially identified in China in January 2020 before rapidly becoming a global pandemic. In an attempt to prevent transmission of the severe acute respiratory syndrome coronavirus disease 2019 (COVID-19) to both health care workers and the community, many hospitals introduced restrictions on non-essential activities within the health care setting. Additional social distancing requirements and restrictions on face-to-face contact in the health care setting and in the general community have been introduced to limit opportunities for COVID-19 transmission. In Australia, a swift emergency response plan to COVID-19 was announced in February 2020 [11] with a staged shift for Australia’s Medicare Benefits Schedule to support the use of telehealth modalities for clinicians (such as obstetricians and midwives) consulting with vulnerable populations including pregnant women and young children [12,13,14,15,16,17,18,19].

Research activities, particularly within potentially vulnerable populations such as pregnant women and young children, were considered non-essential and were thus halted to limit the spread of COVID-19 [20,21]. In order to maintain research momentum in the COVID-19 era, there was a call to find “new” methods to (safely) conduct research [20,21,22,23,24]. Our own research, previously relying on accessing patients in clinical departments and face-to-face data collection, has had to adapt to protect the safety of our participants. We present our traditional and our modified research practices (to comply with COVID-19 restrictions) relating to several studies in various stages of recruitment and outcome assessment. We include a qualitative assessment of considerations and impacts of the modified research practices.

## 2. Study Methods, Adaptations for COVID-19, and Their Implications

At the time of the initial COVID-19 restrictions in Australia in March 2020, the Women and Kids Theme of the South Australian Health and Medical Research Institute (SAHMRI) was conducting a number of studies requiring face-to-face contact and appointments at the Women’s and Children’s Hospital in Adelaide. All were prospective longitudinal studies in various stages of recruitment, including prenatal randomized controlled trials [25,26], postnatal observational studies [27], and a longitudinal follow-up study of a neonatal nutrition intervention [28]. All studies are listed on the Australian and New Zealand Clinical Trials Registry (ACTRN12618000937213, ACTRN12619001511123, ACTRN12620000529943, and ACTRN12612000503820), and all have been reviewed and approved by the Women’s and Children’s Health Network Human Research Ethics Committee (HREC/18/WCHN/42, HREC/19/WCHN/18, HREC/19/WCHN/140, and HREC/17/WCHN/187).

In modifying our research study practices to comply with COVID-19 restrictions, we sought the advice of our SAHMRI Women and Kids Consumer Board, whose members are mostly parents. Our proposed changes involved avoiding hospital appointments in favor of virtual appointments, using electronic consent, and online recruitment services. Feedback from the Consumer Board was positive for all changes. Further suggestions from the board included ensuring that technologies for virtual appointments allowed videoconferencing so as to build rapport with participants. It was also suggested that we endeavor to have one primary study staff member as the contact point for each participant to assist with rapport and continuity of care. The Consumer Board also encouraged the continuation of virtual appointments, even as face-to-face appointments became feasible again once restrictions were eased.

Research activities for recruitment, consent, appointments, and outcome assessments were revised and adapted to comply with COVID-19 restrictions and are summarized in Table 1.

### 2.1. Recruitment

Pregnant women and young children represent a small proportion of the general population that is difficult to reach and target. Direct approach in clinical departments such as at routine antenatal appointments, neonatal special or intensive care units, or postnatal wards has been essential for reaching the target population for observational dietary studies and nutritional intervention trials.

#### 2.1.1. Traditional Methods

Research assistants or research nurses would normally approach families presenting at antenatal and postnatal health care services, explain the study in person, undertake screening via interview, and then obtain consent from participants, or their consent to be contacted at a later date.

#### 2.1.2. Modified Methods

When restrictions on face-to-face activities were introduced, we approached a commercial service provider to undertake a digital media campaign, including online pre-screening, with the approval of the Human Research Ethics Committee. Study advertisements were presented to potential participants on popular social media platforms, such as Google, Instagram, and Facebook who are identified using propriety algorithms specific to each platform. Potential participants are matched with study-specific key demographic criteria, such as approximate geographical location, and their browsing activities, for example searching online for a pregnancy test or pregnancy-related information. Individuals interested in learning more about the study can click on the advertisement and are then taken to an information page. If interested, potential participants can either contact study personnel, or click to complete online self-screening or pre-screening, depending on the requirements for the individual study.

In our questionnaire-based observational studies, self-screened eligible participants can complete e-consent to enroll directly for the study. For interventional trials and studies with invasive procedures (such as blood tests), participants who pass pre-screening can book a (virtual) appointment with study personnel, and, if eligible, undertake the informed consent conversation.

#### 2.1.3. Considerations for, and Implications of, Changed Methodology

To date, this online recruitment strategy has been effective in reaching and recruiting pregnant women to intervention and cohort studies, with opportunities to expand recruitment statewide and/or nation-wide rather than being restricted to a single antenatal care center. Online recruitment has the added benefits of reaching women earlier in pregnancy rather than us having to wait until their first presentation at the health care center. However, our postnatal studies have been more variable in terms of the number of participants enrolling. It is unclear as to whether the target population is seeing the study material or is less inclined to click the links to learn more about the studies and undertake self-screening. Engaging with families of newborn infants who were normally recruited on postnatal wards has been particularly problematic, with very few participants enrolling on a weekly basis, although parents of older infants and toddlers have been more responsive.

### 2.2. Consent

Before enrollment in a research study, participants should provide informed consent as per the Good Clinical Practice Guideline developed by the International Conference on Harmonization [29]. Informed consent requires that the individual, or their legal representative (such as the parent of an infant or child), fully understands the research study and what their involvement will entail, and that any agreement to participate is voluntary [29,30,31]. Written informed consent, with a signature and date from both the participant and research personnel obtaining consent is considered best practice [29,30,31].

#### 2.2.1. Traditional Methods

Previously, we obtained written, hard-copy consent for intervention studies at an enrolment appointment where the study had been verbally explained to them in person and the participant had been provided written information before the appointment, thus allowing them time to consider the study and discuss it with others before they signed the consent form. The research personnel obtaining consent would co-sign the form and photocopy the signed information sheet and the consent form document to provide a copy to the participant. The original hard copy of the signed consent form, along with any other hard copy records would be filed and stored securely onsite for the duration of the study before being archived for storage offsite.

#### 2.2.2. Modified Methods

As face-to-face appointments were no longer feasible, we considered using the postal service to send hard copy consent forms and e-consent options to eligible participants. While hard copy forms sent by post were considered by some to be most similar to our usual methods, the burden on participants required to print the documents and either scan and email them back to the study center or to find a post box to return their signed form (when advised to remain at home), combined with postal service delivery delays, ruled out this option.

The technology for remote or e-consent has been available for some time and is used in survey or questionnaire-based studies. We implemented Adobe Sign and a Research Electronic Data Capture (REDCap) e-consent module to obtain e-signatures from participants for our intervention studies. Both can be accessed through a web browser or mobile device and are compatible with basic smartphones. Potential participants, after passing screening, have a phone call or video conference discussion with staff who explain the study. The participant is afterwards sent the electronic information sheet and consent form to review at their leisure. Then, participants can discuss it further with study staff and/or sign if they wish to participate. Study personnel receive an automated notification to co-sign the e-consent form, and a copy is automatically sent to the participants.

#### 2.2.3. Considerations for, and Implications of, Changed Methodology

Approval for transitioning from hard-copy written consent to e-consent from the overseeing ethics committee required assurance that the standards for obtaining informed consent under Good Clinical Practice guidelines were met [29,31], and additionally that the principles of the Food and Drug Administration’s Part 11 of Title 21 of the Code of Federal Regulations; Electronic Records; Electronic Signatures (21 CFR Part 11) [32] and the Health Insurance Portability and Accountability Act of 1996 [33] were met.

E-consent methods were received positively by participants and study personnel. Anecdotal benefits included the fact that the automated time and date stamps linked to e-signatures ensured that dates were accurate. The only disadvantage reported by study staff was that participants occasionally signed a consent form outside work hours and research staff were unable to co-sign until the next business day, rather than on the same day. E-consent reduces the long-term storage of hard copy documents, with the advantage of being more cost-effective and environmentally sustainable.

### 2.3. Appointments and Outcome Assessments

Outcomes of key interest for nutrition in the first 1000 days include child growth, nutritional status, neurodevelopment, infant and maternal health, pregnancy outcomes, immune outcomes, and the microbiome. Outcome measures often require face-to-face appointments with calibrated equipment and trained research personnel to collect a biospecimen or assess growth or neurodevelopment in a standardized way to ensure continuity in technique across the study.

#### 2.3.1. Traditional Methods

Customarily, most of our study outcomes have been collected in face-to-face appointments at our institutional clinic rooms. Demographic characteristics, health-related behaviors, and the home environment have been recorded via face-to-face interview with study personnel, and any measures of anthropometrics and neurodevelopment, or biological samples had likewise been conducted by study personnel at an appointment.

#### 2.3.2. Modified Methods

With the cancellation of non-essential appointments, we transitioned to virtual appointments wherever possible, and we revised our outcomes and measures. Primary outcome data and procedures were prioritized, while data relating to secondary outcomes or exploratory outcomes were forfeited, or procedures were revised. We embraced videoconferencing and telehealth technologies to conduct virtual, remote appointments.

Data collected via interview were simple to capture using videoconferencing technology, or via a phone call, and were unlikely to become invalidated. Other outcomes and study activities were revised to alleviate the need for a face-to-face appointment. Study products, such as nutritional supplements, were sent to participants by post, rather than being provided at a clinic appointment. Data normally collected by trained study staff with calibrated equipment, such as anthropometric data, were captured through self-reported measures at a virtual appointment, with a preference for measures taken by an alternate health care professional, such as a general practitioner, even if the measurement date fell slightly outside the preferred window. Where possible, we collected source data from participants who took photographs of a health record, such as a pregnancy or infant health form, with their smartphone.

In many instances, primary outcomes, such as blood samples or psychometric child neurodevelopment assessments, could not be collected remotely. Whilst all face-to-face research was necessarily paused for a time, it is worth noting that prompt restrictions in Australia have so far prevented a widespread outbreak of COVID-19, and resuming face-to-face assessments has been deemed relatively safe. Where face-to-face assessments were crucial for the study, participants completed COVID-19 screening (such as for symptoms or reported encounters with potentially infected individuals) the day before and again at the time of the appointment. Strict hygiene procedures were followed, the duration of appointments was limited, clinic space for conducting appointments outside the hospital was offered, and staff wore personal protective equipment. While we would previously offer home visits for participants unable to attend a clinic appointment, under COVID-19 restrictions, home visits were suspended.

For studies that required face-to-face contact to collect outcome data, restrictions resulted in delays for appointments that were planned for a specific week of gestation or age of a child and hence led to substantial variation in the timing of outcome assessment. Standardized psychometric tests, such as measuring intelligence quotient, require a face-to-face appointment with an assessor. We ceased our psychometric tests for several months and widened our target age bracket for accepting assessments. As the psychometric test we used is age-standardized, the increased heterogeneity in age at the time of assessments will not preclude the data being meaningful. However, for other outcomes, substantial variation in the timing of assessment may limit the usability of the data.

#### 2.3.3. Considerations for, and Implications of, Changed Methodology

Although our Consumer Board recommended the use of video conferencing when conducting virtual appointments with participants, we found that most participants preferred, and requested, phone calls without video. Virtual appointments were best received when booked in advance with participants for a specific time and day, as would normally occur for an appointment at the hospital. Additional contact points, including text messages, phone calls, and additional study newsletters were added in some studies shortly after enrollment, with the aim of establishing and maintaining rapport. With these additional contact points, study personnel reported no apparent disadvantage in establishing a relationship with participants in the absence of face-to-face appointments.

Importantly, all modifications to data collection procedures during COVID-19 need to be carefully documented, including how data were measured, and by whom, and why a variable is missing. Changes to outcome (or exposure or confounder) measurement techniques can affect the result of the measure, and hence the reliability of any study results, through inconsistent methodology, inaccuracy, measurement error or bias, and misclassification [30,34]. In particular, accepting self or parent-reported outcomes and measures introduces bias as well as measurement variability. For example, it is not possible to train all parents to perform standardized measures of infant length or skin folds, even if calibrated equipment could be provided. Self-rated outcomes are subject to individual differences in interpretation of a question and perception of the outcome of interest and hence are likely to be subject to bias, particularly for dietary assessments. 

## 3. Discussion

Our strategies for continuing clinical research during and post-COVID-19 have involved developing online recruitment and e-consent, virtual appointments, sending study products via the postal service, and redesigning the collection of data for exposure variables and secondary outcomes. Studies in other vulnerable populations, such as cancer patients, have similarly reported implementing the collection of remote informed consent [22,23,35], virtual appointments, and revising the collection of data for secondary and exploratory analyses [22,35].

Others recommendations for conducting face-to-face research in the era of COVID-19 include providing support to avoid public transport if face-to-face appointments are critical [20], accelerating a study to finish sooner [22], and redesigning studies to be less labor intensive [22,24], for example, reducing the number of samples needed [22]. Limiting the collection of outcomes that require face-to-face contact by prioritizing outcomes and data collection [20] is encouraged, bearing in mind that important opportunities will likely be lost, such as those for prospective longitudinal research [22,23]. However, primary outcome measures are of greatest importance [36] and should be prioritized over other, potentially competing, outcome or confounding measures.

There is also a strong urge to conduct research remotely, such as by mailing out study medications or interventions directly to participants [21,22,37], implementing remote surrogate data collection methods [20], for example providing participants with digital tools for measuring their own blood pressure, heart rate, or temperature [20], and providing follow-up care and research testing at home or locally if virtual contact is not possible [37] to avoid visits to the primary care setting [20]. We further strongly encourage the ongoing development of psychometric tests that can be administered remotely using telehealth platforms as well as technologies to allow remote assessment of biological markers, such as dried blood spots [38,39,40] and dried breastmilk spots [41].

One critical factor in facilitating the adaption of our research methodologies quickly has been the increased review and support from the governing ethics committee, the Women’s and Children’s Hospital Human Research Ethics Committee. Support from the ethics committee has been crucial for limiting the cessation of research practices. Other Australian researchers have likewise noted shortened time frames for ethics committee procedures, thus allowing research to adapt rapidly in line with current (COVID-19 era) requirements [42].

Adaptations such as the above recommendations and those that we have adopted are necessary to maintain ongoing and new research to advance science in the face of COVID-19 [20] and to foster the next generation of early-career researchers [21,42]. However, many of these adaptations and technological advances will be useful to maintain even once the threat of COVID-19 has passed [21]. Support for virtual appointments to replace many face-to-face appointments has been widespread in both the research [21,22,35,37] and the primary health care [12,13,14,15,16,17,18,19] communities. The technology for virtual appointments and e-consent has been possible for some time. Previously, there have been barriers to the adoption of telehealth and telemedicine within the primary care setting from both clinicians, health care systems, and governing bodies [15,16]. These include the requirement for clinicians to adapt to new methods of consulting, the perception that virtual appointments are not effective and safe, a lack of awareness of telehealth technologies, the absence of telehealth in the training of health care providers, a perceived erosion of the patient–clinician relationship, and obtaining appropriate renumeration from patients and/or insurance or health care systems [15,16]. These barriers have likewise limited the uptake of digital technologies by both researchers and human research ethics committees.

### 3.1. Considerations

Whilst the modifications to the study methodology detailed here are still relatively new, we have discussed several important implications to be taken into consideration if implementing any of these changes. Virtual trials, with no physical sites or face-to-face contact with participants, are now possible, as are hybrid and/or decentralized trials, using telemedicine and remote technologies where possible, complemented by home visits or local community facilities for necessary face-to-face procedures. Self (or parent) reported outcomes and measures (such as finger-prick blood spot samples) have the advantage of allowing studies to be conducted entirely virtually. Digital technologies can include remotely located participants, thus broadening the pool of potential participants, rather than limiting it to one health care center. In many instances, expanding recruitment nationwide or even internationally may allow recruitment to be completed in a much shorter timeframe. It also permits the inclusion of participants for whom travel to an appointment is inadvisable, as well as limiting unnecessary contact with the primary care setting [12,13,15,16,17,18,19]. Electronic consent is more environmentally sustainable, and digital forms are easier to store and more easily accessed at a later date than hard copies. However, there are important disadvantages for conducting studies remotely. Objective face-to-face outcome assessment replaced by subjective self-report or parent-report questionnaires will be less accurate and potentially subject to bias. Measures conducted by individual participants will have increased variability and less reliable data. Careful consideration is important for the interpretation of studies with subjective outcome assessment, particularly where some data have been collected through objective outcome assessment by trained research staff. We recommend that sensitivity analyses be used to explore the effect of subjective assessments on study results.

The COVID-19 pandemic has resulted in a rapid embrace and uptake of these technological advances in both the health care and research settings. Virtual appointments not only protect participants but research staff as well, allowing the research workforce to work from home where necessary [20,43]. Likewise, primary care provisions in countries such as Australia have implemented virtual appointments with health care providers wherever possible to avoid face-to-face contact and limit the risk of exposure to the health care workforce [12,13,14,15,16,17,18,19]. Given the new technologies available and their successful implementation in our own program of research, we endorse their further use (with some reservations) in conducting research remotely both for participants and research staff.

### 3.2. Strengths and Limitations

While the focus of the (limited) literature to date on conducting research during COVID-19 has been on research in cancer patients who require ongoing medical care [18,22,23], we here discuss our experience across multiple studies in a population that is vulnerable for various reasons. Furthermore, we undertook consumer engagement when changing our study activities to gauge acceptance and canvas opportunities to improve participant experience. However, it is important to note that our use of technologies for conducting research remotely is still in its early stages. There are likely to be other opportunities and strategies not explored here. There may be further benefits or disadvantages not yet discovered, and it is likely that the increased uptake of modern technologies will promote further advances that build on these benefits and open the way for new developments. It will be important to explore whether the differing recruitment or data capture strategies have any effects on the participant characteristics [44,45,46], primary outcomes, or conclusions of each study [34,36,47]. It is imperative that any changes to study design, methodology, or methods of data collection are well defined and documented as the studies progress [36].

## 4. Conclusions

COVID-19 brings a new opportunity to embrace new technologies and maintain them in the future, both for rural patients and for vulnerable populations, as well as to avoid unnecessary travel and its burden on individuals and the environment. Critical limitations of and biases potentially introduced by some modifications need to be carefully considered when implementing changes to study procedures and interpreting study results. As we continue our rapid adaptation to the changing circumstances, it is important to share strategies, as well as our successes and failures, with fellow researchers.

## Figures and Tables

**Table 1:** Methodology of maternal, infant, and child nutrition studies prior to coronavirus disease 2019 (COVID-19), and amendments in the era of COVID-19.

	Research Methodology		
Research Activity	Original	Amendment(s)	Implications
Recruitment	Hospital face-to-face screening and recruitment while women attended routine antenatal visit, or while women were patients on the postnatal ward	Online recruitment through digital media campaign, self-referral following pre-screening (with a potential follow-up phone call screening with study staff)	Pro: recruitment expanded statewide–nationwide rather than restricted to an individual center Pro: women identified earlier in pregnancy than would traditionally present at antenatal care settings Pro: effective recruitment of pregnant women Con: ineffective treatment of families with newborn infants
Consent	Hard copy written informed consent at time of enrolment appointment	e-consent (Research Electronic Data Capture and Adobe Sign)	Pro: automated time and date stamps minimize human error Pro: improved environmental sustainability through avoiding printing and storing hard-copy documents Con: participants occasionally completed the consent outside of business hours so that research staff co-signing does not occur on the same day
Appointment and outcome assessment	Face-to-face interview with study staff via clinic appointment at hospital	Telephone call/virtual interview with study staff	Pro: both participants and research staff can conduct interviews from home Con: possibly more difficult to maintain rapport
	Study intervention product provided at clinic appointment at hospital	Study product posted to participants residential address	Con: delay between enrollment and commencement of study intervention
	Face-to-face outcome measures (secondary and exploratory) conducted by study staff via clinic appointment at hospital	Virtual appointment with self-reported measures, revised secondary outcomes	Con: not objective (subject to differences in perceptions and interpretations) Con: possible measurement error Con: uncalibrated equipment Con: missing outcomes Con: possible bias
	Face-to-face outcome measures (primary) conducted by study staff via clinic appointment at hospital	Face-to-face appointment with study staff with delayed primary outcome measures, use of personal protective equipment, shorter appointments, increased hygiene and COVID-19 screening	Con: differences in outcome assessment procedures before versus during COVID-19 Con: missed non-primary outcome data Con: wider variation in age and timing of assessment, possibly precluding meaningful combination and interpretation of data

## Data Availability

No data is presented in this manuscript.
